# Screening of Pandemic Response Box Library Reveals the High Activity of Olorofim against Pathogenic *Sporothrix* Species

**DOI:** 10.3390/jof8101004

**Published:** 2022-09-25

**Authors:** Luana Pereira Borba-Santos, Rodrigo Rollin-Pinheiro, Yasmin da Silva Fontes, Giulia Maria Pires dos Santos, Glauber Ribeiro de Sousa Araújo, Anderson Messias Rodrigues, Allan J. Guimarães, Wanderley de Souza, Susana Frases, Antonio Ferreira-Pereira, Eliana Barreto-Bergter, Sonia Rozental

**Affiliations:** 1 Laboratório de Biologia Celular de Fungos, Instituto de Biofísica Carlos Chagas Filho, Universidade Federal do Rio de Janeiro, Rio de Janeiro 21941-902, Brazil; 2 Laboratório de Química Biológica de Microrganismos, Departamento de Microbiologia Geral, Instituto de Microbiologia Paulo de Góes, Universidade Federal do Rio de Janeiro, Rio de Janeiro 21941-902, Brazil; 3 Laboratório de Bioquímica Microbiana, Departamento de Microbiologia Geral, Instituto de Microbiologia Paulo de Góes, Universidade Federal do Rio de Janeiro, Rio de Janeiro 21941-902, Brazil; 4 Laboratório de Biofísica de Fungos, Instituto de Biofísica Carlos Chagas Filho, Universidade Federal do Rio de Janeiro, Rio de Janeiro 21941-902, Brazil; 5 Laboratório de Patógenos Fúngicos Emergentes, Departamento de Microbiologia, Imunologia e Parasitologia, Universidade Federal de São Paulo, São Paulo 04023-062, Brazil; 6 Laboratório de Bioquímica e Imunologia das Micoses, Departamento de Microbiologia e Parasitologia, Instituto Biomédico, Universidade Federal Fluminense, Niterói 24210-130, Brazil; 7 Laboratório de Ultraestrutura Celular Hertha Meyer, Instituto de Biofísica Carlos Chagas Filho, Universidade Federal do Rio de Janeiro, Rio de Janeiro 21941-902, Brazil

**Keywords:** antifungal development, dimorphic fungi, antifungal target, sporotrichosis, olorofim, yeast, biofilm, Medicines for Malaria Venture

## Abstract

The increase in the prevalence and severity of fungal infections and the resistance to available antifungals highlights the imperative need for novel therapeutics and the search for new targets. High-content screening of libraries containing hundreds of compounds is a powerful strategy for searching for new drug candidates. In this study, we screened the Pandemic Response Box library (Medicines for Malaria Venture) of 400 diverse molecules against the *Sporothrix* pathogenic species. The initial screen identified twenty-four candidates that inhibited the growth of *Sporothrix brasiliensis* by more than 80%. Some of these compounds are known to display antifungal activity, including olorofim (MMV1782354), a new antifungal drug. Olorofim inhibited and killed the yeasts of *S. brasiliensis*, *S. schenckii*, and *S. globosa* at concentrations lower than itraconazole, and it also showed antibiofilm activity. According to the results obtained by fluorimetry, electron microscopy, and particle characterization analyses, we observed that olorofim induced profound alterations on the cell surface and cell cycle arrest in *S. brasiliensis* yeasts. We also verified that these morphophysiological alterations impaired their ability to adhere to keratinocytes in vitro. Our results indicate that olorofim is a promising new antifungal against sporotrichosis agents.

## 1. Introduction

Annually, fungal infections are responsible for numerous deaths worldwide [1]. Among the limitations presented by the antifungal agents used for systemic treatment, we can mention: (i) suboptimal efficacy; (ii) toxicity; (iii) drug interactions; and (iv) the emergence of resistant species [2]. The increasing incidence of fungal infections highlighted the need to find new treatment alternatives [1].

It is essential to study more effective drugs, which act more selectively on the fungal cell and require shorter treatment and to even explore compounds that act on distinct cellular targets rather than those of commercial antifungals, thus preventing the development of cross-resistance. An alternative strategy is to search for new antifungal candidates from available molecule libraries [3]. In this study, we used the Pandemic Response Box library, developed by the Medicines for Malaria Venture (MMV) organization, which contains 400 molecules with known activity against fungi, bacteria, and viruses [4]. Some of these molecules are commercially available drugs, while others are new compounds that showed promising biological activities.

Dimorphic fungi are less sensitive to antifungal treatment, which usually requires a long therapeutic regimen [5]. These include sporotrichosis, the most common human subcutaneous mycosis globally, which also affects animals, especially cats [6]. Sporotrichosis can be acquired as sapronosis or zoonosis, and zoonotic transmission is the main form observed in Brazil, with the species *Sporothrix brasiliensis* being the most frequent [6]. Zoonotic sporotrichosis, currently endemic in Brazil and causing hundreds of cases, is expanding to other Latin American countries [7,8].

There are a few options for sporotrichosis treatment, with itraconazole as the first choice. However, the treatment is lengthy and expensive and promotes considerable side-effects for patients (gastrointestinal and hepatotoxicity, for example) [9]. In addition, therapeutic failures and identifying isolates with reduced sensitivity to itraconazole are increasingly frequent in Brazil [7].

The main objective of our study was to evaluate the Pandemic Response Box library compounds against the medically relevant main causative agents of sporotrichosis: *S. brasiliensis*, *Sporothrix globosa*, and *Sporothrix schenckii*. In addition, we assessed the effects of the most promising compound on yeasts, its selectivity, and the interaction between keratinocytes and treated yeasts.

## 2. Materials and Methods

### 2.1. Isolates and Culture Conditions

The reference isolates *S. brasiliensis* ATCC MYA 4823, *S. schenckii* ATCC 32286, and *S. globosa* CBS 130104 were used in this study. Isolates were kept in a saline solution containing 10% glycerol and 10% glucose at −20 °C. *S. brasiliensis* and *S. schenckii* were initially cultivated in the mycelial form in Sabouraud broth ( BD Difco™, Franklin Lakes, NJ, USA), and an aliquot containing 10^5^ CFU/mL was inoculated into brain heart infusion broth (BD Difco™) supplemented with 2% glucose (pH 7.8) for the conversion to the yeast phase. Both were incubated at 36 °C, with orbital shaking (150 rpm) for 7 days. *S. globosa* was initially cultivated in Sabouraud agar (BD Difco™) at 25 °C for 7 days and converted to the yeast phase by a successive passage on brain heart infusion agar (BD Difco™) at 35 °C in a 5% CO_2_ atmosphere for 7 days. The parasitic yeast phase was used in all assays.

### 2.2. Cells Line and Culture Conditions

Macrophage cell line (RAW 264.7) and keratinocyte cell line (HaCat) were maintained in DMEM medium (Sigma-Aldrich®, San Luis, MO, USA) with a pH 7.2 supplemented with 10% fetal bovine serum (BD Difco™) in 5% CO_2_ atmosphere at 37 °C.

### 2.3. Compounds

Medicines for Malaria Venture (MMV, Geneva, Switzerland) kindly provided the Pandemic Response Box library containing 400 compounds diluted in dimethyl sulfoxide (DMSO) at 10 mM [4]. All compounds were diluted to 1 mM in DMSO and stored at −20 °C until use. Additional experiments were conducted using olorofim powder, also provided by MMV. Itraconazole (1 mM, Sigma-Aldrich®) in DMSO was used as a standard antifungal.

### 2.4. Screening the Pandemic Response Box

The reference isolate *S. brasiliensis* ATCC MYA 4823 was used to screen the Pandemic Response Box compounds for inhibitors of fungal growth. Compounds were diluted in supplemented RPMI medium (RPMI 1640 supplemented with 2% glucose and buffered with 0.165 M MOPS to pH 7.2) (Sigma-Aldrich®) in flat-bottom 96-well microplates to a final concentration of 1 µM, following the MMV recommended guidelines for screening [10]. Yeasts were counted in a hemocytometer, and a standardized yeast suspension of 1 × 10^5^ CFU/mL was used in experiments. Yeasts were added to each well, and the microplate was incubated for 48 h at 35 °C in a 5% CO_2_ atmosphere. After 48 h, fungal growth was analyzed by visual inspection using an inverted light microscope (Axiovert 100, ZEISS, Oberkochen, Germany) and quantified by spectrophotometric readings at 492 nm using a microtiter plate reader (EMax Plus, Molecular Devices, San José, CA, USA). The absorbance value for each well was subtracted from the value for the negative control (well containing only supplemented RPMI media). Inhibition of fungal growth (I) relative to untreated controls was calculated using the following equation: I = 100 − (A × 100/C), where A is the absorbance of treated wells, and C is the absorbance of untreated wells. As an additional control, 0.1% DMSO solution was included in all experiments. Inhibitions of more than 80% were defined as the cut-off to select the most promising compounds, corresponding to clearly visible growth prevention when samples were initially analyzed by visual inspection. Results are representative of three independent experiments performed in duplicate.

### 2.5. Determination of Minimum Inhibitory Concentration

The broth microdilution technique, adapted for *Sporothrix* cells, was used to determine the minimum inhibitory concentration (MIC) values of the most active compounds [11]. Briefly, serial 2-fold dilutions of the compounds were prepared in supplemented RPMI in flat-bottom 96-well microplates to obtain a final concentration ranging from 0.002 to 1 µM (except for luliconazole, whose final concentration ranged from 1 to 0.0002 µM). Yeasts were added to microplates at a final concentration of 1 × 10^5^ CFU/mL and incubated at 35 °C for 48 h in a 5% CO_2_ chamber. Fungal growth was analyzed by visual inspection using an inverted light microscope (Axiovert 100, ZEISS) and quantified by spectrophotometric readings at 492 nm (Emax Plus plate reader, Molecular Devices). Negative controls were included in experiments for the subtraction of absorbance values. Concentrations that inhibit at least 50% of fungal growth (IC_50_) were calculated using the following equation: I = 100 − (A × 100/C), where A was the absorbance of treated wells, and C was the absorbance of untreated wells. Results are presented as the mean of two independent experiments performed in duplicate.

### 2.6. Killing Assay

Yeasts (10^5^ CFU/mL starting inoculum) were exposed to distinct concentrations of olorofim or itraconazole (0, 1 µM, 10 µM, 50 µM, and 100 µM) in supplemented RPMI for 48 h at 35 °C in a 5% CO_2_ atmosphere. Samples were homogenized, and 10 µL were plated on BHI agar and incubated for 5 days at 35 °C in a 5% CO_2_ atmosphere. Images of agar plates were digitalized and processed using ImageJ 1.52a software (https://imagej.nih.gov/ij/, accessed on 19 September 2022 ), and fungal growth was quantified regarding the total area of the colonies. Results are presented as the mean of two independent experiments performed in duplicate.

### 2.7. Effect of Olorofim on Mature Biofilms

To obtain *Sporothrix* biofilms, yeast suspensions (10^6^ CFU/mL) in supplemented RPMI were transferred into 96-well microplates (100 µL) (Corning Inc., New York, NY, USA) and incubated for 5 days at 35 °C and 5% CO_2_. After removing the supernatant, 100 µL of olorofim was added to final concentrations ranging from 1 to 64 µM in supplemented RPMI. The same concentrations of itraconazole were tested in parallel. Biofilms were incubated for 48 h (at 35 °C and 5% CO_2_), and the metabolic activity was quantified using the XTT reduction assay, with color change measured by spectrophotometric readings at 492 nm (Emax Plus plate reader, Molecular Devices). Inhibition of biofilm metabolic activity was calculated according to the following equation: I = 100 − (A × 100/C), where A is the absorbance of treated biofilms, and C is the absorbance of untreated biofilms. Results are representative of three independent experiments performed in duplicate.

### 2.8. Fluorimetry Assays

*S. brasiliensis* yeasts (1 × 10^5^ CFU/mL) were incubated with 0.015, 0.03, and 0.06 µM of olorofim in supplemented RPMI for 48 h at 36 °C in an orbital shaker. Untreated controls were grown in the absence of drugs. Cells were washed in sterile saline, fixed in 2% formaldehyde, counted, and 1 × 10^7^ cells were incubated for 30 min at room temperature in the dark with the following fluorochromes: 20 μM SYTOX™ Blue; 25 μg/mL concanavalin A conjugate with Alexa Fluor™ 488 (Thermo Fisher Scientific, Waltham, Massachusetts, USA); 30 μg/mL aniline blue; or 25 μg/mL calcofluor white (Sigma-Aldrich®). Samples were washed in saline. Yeast suspensions were standardized again, and 100 µL/well was added to a 96-well clear-bottom dark-side microplate. The fluorescence intensity was measured using a Spectra-MAX 340 microplate reader (Molecular Devices), according to the following parameters: 480 nm (excitation at 444 nm) for SYTOX Blue; 519 nm (excitation at 495 nm) for concanavalin A; 502 nm (excitation at 398 nm) for aniline blue; and 432 nm (excitation at 350 nm) for calcofluor white. Results are representative of three independent experiments performed in triplicate.

### 2.9. Zeta Potential (ζ) and Conductance

*S. brasiliensis* yeasts were treated as described, washed, and resuspended with pyrogenic water, and 1 × 10^6^ cells were used to measure the zeta potential and conductance in a zeta potential analyzer (NanoBrook Omni particle, Brookhaven Instruments Corporation, Holtsville, NY, USA) at 25 °C. Twenty measurements were performed in each experiment. Results are representative of three independent experiments [12].

### 2.10. Scanning Electron Microscopy

Untreated or treated yeasts with 0.06 µM of olorofim were washed in sterile saline and fixed in 2.5% glutaraldehyde and 4% formaldehyde in 0.1 M cacodylate buffer for 1 h. Cells were washed in 0.1 M cacodylate buffer, adhered to poly-L-lysine-coated (Sigma-Aldrich®) glass coverslips, and post-fixed with 1% osmium tetroxide in 0.1 M cacodylate buffer containing 1.25% potassium ferrocyanide for 30 min. Samples were dehydrated in a graded ethanol (Merck KGaA, Darmstadt, Germany) series, critical-point-dried in CO_2_ (EM DPC 300, Leica, Wetzlar, Germany), and coated for sputter with gold/palladium (Au/Pd) (Balzers Union sputtering device FL-9496, Balzers Union, Balzers, Liechtenstein). Images were obtained using a Carl Zeiss Evo LS 10 scanning electron microscope (ZEISS) and processed using Photoshop software (Adobe, San José, CA, USA).

### 2.11. Transmission Electron Microscopy

Untreated and treated yeasts with 0.06 µM of olorofim were washed in sterile saline and fixed overnight (at 4 °C) in 2.5% glutaraldehyde and 4% formaldehyde in 0.1 M cacodylate buffer. Cells were washed in 0.1 M cacodylate buffer, post-fixed with 1% osmium tetroxide in 0.1 M cacodylate buffer containing 1.25% potassium ferrocyanide for 2 h (at 4 °C), and dehydrated in a graded ethanol series (30, 50, 70, 90, and 100% for 30 min each step at 4 °C). Samples were embedded in Spurr resin, and ultrathin sections were stained in uranyl acetate and lead citrate. Images were obtained using a JEOL 1200 EX transmission electron microscope (JEOL, Akishima, Tokyo, Japan) and processed using Photoshop software (Adobe). The cell wall thickness of 100 cells was measured using ImageJ 1.52a software (https://imagej.nih.gov/ij/, accessed on 19 September 2022).

### 2.12. Cytotoxicity Assays

Cytotoxicity assays with olorofim were performed using the keratinocyte cell line HaCaT and the macrophage cell line RAW 264.7. Cells were treated with different concentrations of olorofim ranging from 0.1 to 100 μM and diluted in DMEM medium supplemented with 10% fetal bovine serum. After 48 h of incubation at 37 °C and 5% CO_2_, concentrations that elicited 50% cytotoxicity (CC_50_) were estimated according to the Neutral Red assay. The selectivity towards *Sporothrix* spp. was determined using the median of MIC values previously obtained. The selectivity index (SI) of olorofim was calculated using the following equation: SI = CC_50_/MIC median. Experiments were also performed in parallel with itraconazole as a control. Results are representative of three independent experiments performed in triplicate.

### 2.13. Interaction between Keratinocytes and Treated Yeasts

HaCaT cells (5 × 10^5^ cells) were seeded in 24-well plates with glass coverslips in DMEM medium supplemented with 10% fetal bovine serum and incubated for 48 h at 37 °C and 5% CO_2_. Before the interaction assay, untreated and treated yeasts with 0.06 µM of olorofim or 0.125 µM of itraconazole were washed in sterile saline, and 1 × 10^7^ cells were resuspended in DMEM medium. Keratinocytes were exposed to *S. brasiliensis* yeasts at a 5:1 ratio (fungus: cell) for 24 h. After incubation, the coverslips were washed with sterile saline and fixed with 4% formaldehyde (Sigma-Aldrich®) for 20 min. The coverslips were stained with Giemsa (Sigma-Aldrich®) for 1 h. Finally, these coverslips were washed in serial solutions of acetone and xylene (Sigma-Aldrich®) and adhered to the glass slide. The interaction rate was calculated using the following equation: Ir = A/B, where A is the number of keratinocytes with yeasts adhered to their surface after 24 h, and B is the total keratinocytes [13]. The interaction was quantified by counting 100 keratinocytes in each coverslip. Results are representative of three independent experiments performed in duplicate.

### 2.14. Statistical Analyses

Statistical analysis was performed using Prism 9 software (GraphPad Software, San Diego, CA, USA), and *p* < 0.05 was considered statistically significant. Differences in fluorescent intensity, physicochemical properties, and interaction ratio were analyzed by one-way analysis of variance (with Dunnett’s *post hoc* test), while the student *t*-test (Mann–Whitney test) was used to examine differences in the cell wall thickness.

## 3. Results

### 3.1. The Most Promising Molecules from the Pandemic Response Box Library Were Known Antifungals

**Figure 1:**
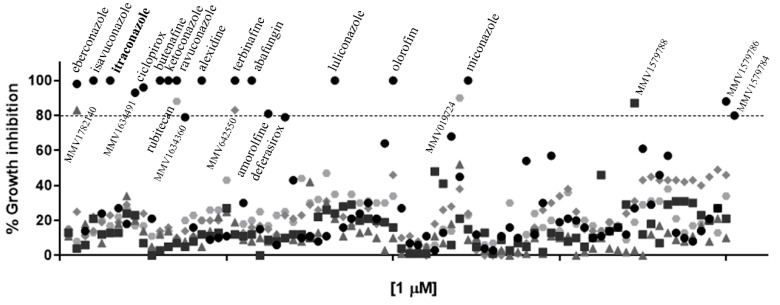
Growth inhibition of *Sporothrix brasiliensis* yeasts after exposure to the 400 compounds of the Pandemic Response Box library for 48h. Itraconazole (in bold) is the antifungal recommended for sporotrichosis treatment. Results are shown as the mean of percent growth relative to the untreated control.

**Figure 2:**
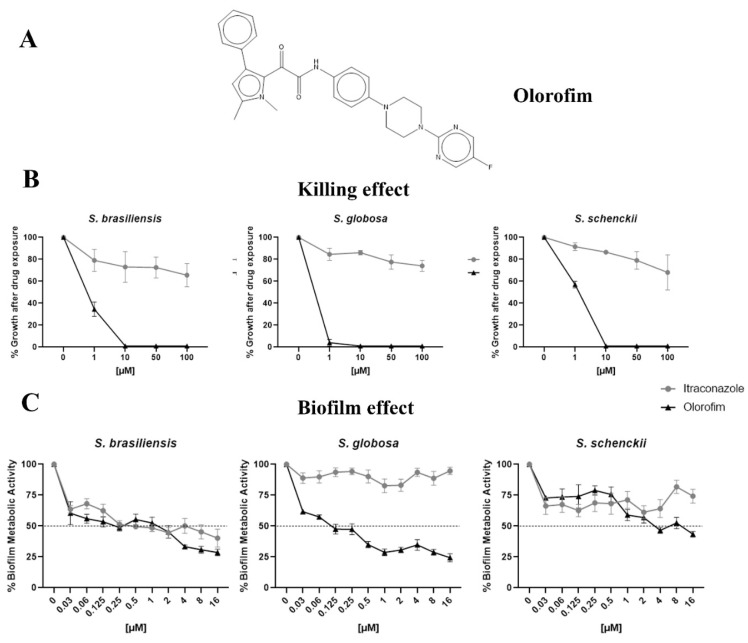
The anti-*Sporothrix* activity of olorofim. The chemical structure of olorofim (**A**). After exposure to olorofim or itraconazole, the yeast killing was evaluated by cultivation in a drug-free medium (**B**). The ability of olorofim to inhibit mature biofilms was determined according to the metabolic activity of cells in the presence of compounds (**C**). Results are shown as the mean of percent growth or metabolic activity and the standard error of the mean.

Among the 400 compounds screened from the Pandemic Response Box library, twenty-four were able to inhibit at least 80% of *S. brasiliensis* growth at 1 µM (Figure 1 and Supplementary Table S1). Besides itraconazole, we identified thirteen known antifungals and two new molecules with previously described antifungal activity, in addition to six antibacterial and three antiviral compounds. Microdilution assays confirmed the inhibitory actions of these compounds against *S. brasiliensis* yeasts; however, some drugs were not active against *S. globosa* and *S. schenckii* up to 1 µM (Table 1). Luliconazole (MMV1782224) and olorofim (MMV1782354) showed the lowest MIC, with values lower or equal to itraconazole (Table 1). Luliconazole was not selected for further experiments due to its considerable cytotoxicity [14]. We selected olorofim (Figure 2A) for further studies exploring its effects on *Sporothrix* yeasts due to its distinct mechanism of action based on the inhibition of pyrimidines biosynthesis [15].

### 3.2. Olorofim Killed Yeasts and Exhibited Antibiofilm Activity at Concentrations Lower than Itraconazole

Olorofim was fungicidal to *Sporothrix* yeasts at 10 µM. In contrast, itraconazole could not kill these species in concentrations lower than 100 µM (Figure 2B). On the other hand, olorofim inhibited about 50% of *S. brasiliensis* and *S. globosa* biofilms at 0.125 µM, but a higher concentration (4 µM) was necessary to obtain a similar result against the *S. schenckii* biofilm. Itraconazole could not inhibit *S. globosa* or *S. schenckii* biofilms up to 16 µM (Figure 2C).

### 3.3. Olorofim Was Highly Selective towards Sporothrix Cells

Olorofim did not elicit 50% cytotoxicity of HaCaT or RAW 264.7 cells after 48 h of exposure at concentrations ranging from 0.1 to 100 µM, similar to itraconazole (Table 2). The estimated selectivity index (MIC_median_/CC_50_) indicates that olorofim is at least 1666.7 times more selective towards *Sporothrix* cells than keratinocytes or macrophages (Table 2).

### 3.4. Olorofim Led to DNA Accumulation and Superficial Changes in S. brasiliensis Yeasts

**Figure 3:**
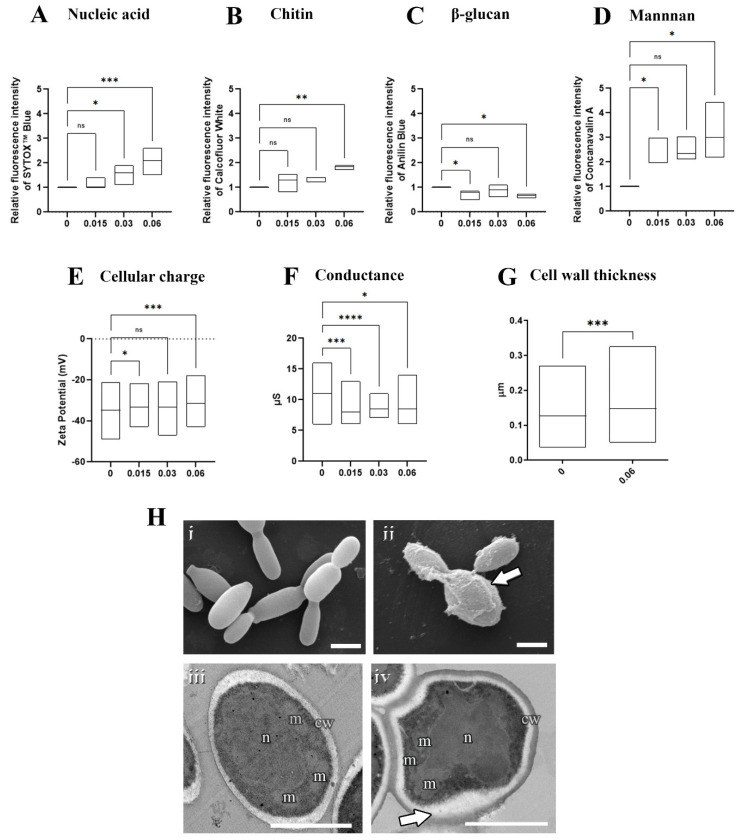
Effects of olorofim exposure on *S. brasiliensis* yeasts. Treated yeasts analyzed by fluorimetry exhibited a dose−dependent DNA accumulation (**A**), increased chitin (**B**), decreased β−glucan (**C**), increased mannan (**D**), and reduced electronegativity (**E**) and conductance (**F**). Cell wall measurements revealed that olorofim treatment increased the thickness of this structure (**G**). Scanning electron microscopy images showed untreated yeasts with an elongated shape (**Hi**), and treated cells with alterations in cell wall integrity, exhibiting a granular appearance (arrow in **Hii**). Transmission electron microscopy images illustrated untreated cells with homogeneous cytoplasm with nucleus (n) and mitochondria (m), surrounded by a plasma membrane and cell wall (cw) (**Hiii**), while treatment with olorofim induced an amorphous shape and modifications in the cell wall structure (arrow in **Hiv**). Graphs showed the min and maximum with the line in the median, with the *X*−axis representing olorofim concentrations in µM. * *p* < 0.05, ** *p* < 0.01, *** *p* < 0.001, **** *p* < 0.0001, ns: not significant by one-way ANOVA. Scale bars: 2.5 µm (**Hi**,**Hii**) and 0.5 µm (**Hiii**,**Hiv**).

To assess the mechanism of the action of olorofim on *Sporothrix* cells, *S. brasiliensis* yeasts were exposed to 0.015 µM (MIC/4), 0.03 µM (MIC/2), or 0.06 µM (MIC), followed by analysis using fluorimetry, microscopy, and particle characterization (Figure 3). The fluorometric analyses revealed that olorofim induced DNA accumulation at concentrations higher than 0.03 µM (Figure 3A) and changes in the cell wall composition, with increased chitin and mannan contents and decreased β-glucan at 0.06 µM (Figure 3B–D). In addition, olorofim-treated cells were less electronegative than untreated yeasts and presented lower conductance, indicating that changes in the cell wall composition alter the cellular charge and surface properties (Figure 3E,F). The scanning and transmission electron microscopy confirmed that the cell wall structure was profoundly changed after treatment with 0.06 µM olorofim (Figure 3H). The treated cell wall exhibited a granular appearance (arrow in Figure 3(Hii) and increased thickness (Figure 3G,(Hiv)).

### 3.5. The Ability of S. brasiliensis Yeasts to Adhere Keratinocytes Decreased after Olorofim Exposure

**Figure 4:**
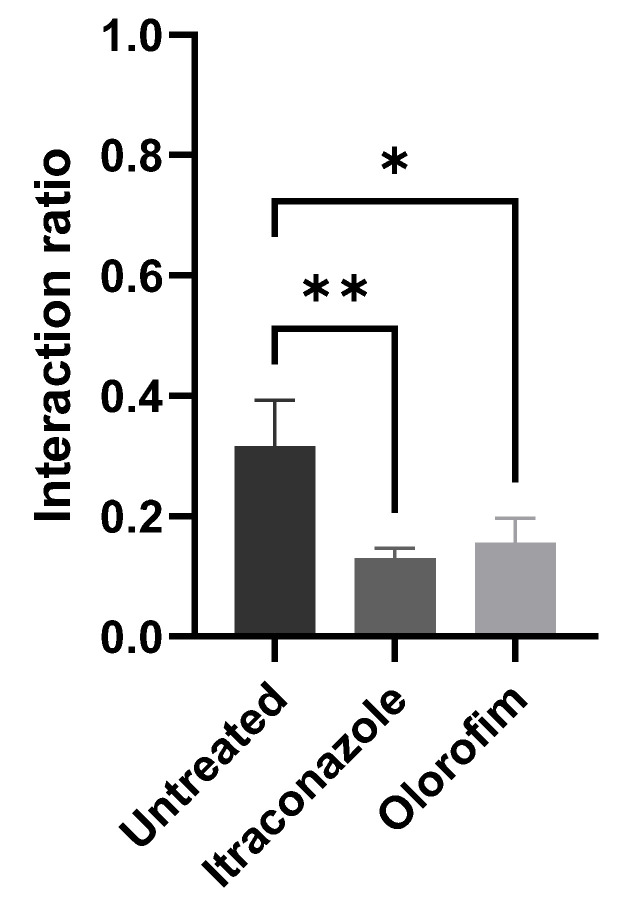
Interaction between keratinocytes and *S. brasiliensis* yeasts. Yeasts treated with MIC of compounds (0.06 µM olorofim or 0.125 µM itraconazole) displayed decreased ability to adhere to the surface of keratinocytes. * *p* < 0.05 and ** *p* < 0.01 by one-way ANOVA.

After 24 h of interaction between HaCaT and *S. brasiliensis* yeasts (with previous exposure to MIC of olorofim or itraconazole), we quantified the number of keratinocytes with yeasts adhered to their surface. We observed that exposure to olorofim decreased the ability of yeast to interact with keratinocytes (Figure 4). Treatment with itraconazole induced a more significant reduction in the interaction rate; however, the concentration at which the yeasts were exposed was 0.125 µM (MIC), corresponding to twice the olorofim concentration.

## 4. Discussion

Sporotrichosis was recently added to the list of Neglected Tropical Diseases by the World Health Organization, and efforts to understand its pathogenic agent have increased in recent years [7,16]. Here, we evaluated the anti-*Sporothrix* activity of 400 compounds from the Pandemic Response Box library and demonstrated the high efficacy of olorofim, a new antifungal that belongs to the orotomide class.

The initial screening showed that twenty-four compounds from the Pandemic Response Box library have in vitro activity against *S. brasiliensis* (Figure 1). Some of these compounds are commercial antifungals used in topical posology to treat fungal infections (abafungin, amorolfine, butenafine, ciclopirox, deferasirox, eberconazole, ketoconazole, luliconazole, miconazole, and terbinafine). Itraconazole and terbinafine presented in the library served as internal controls, confirming the validity of our screening technique because both antifungals are recommended for sporotrichosis treatment and exhibit low MIC values [7].

Launched in 2005, luliconazole is an azole for the topical treatment of *Tinea pedis*, candidiasis, and pityriasis versicolor [14]. This compound exhibited the lowest MIC values during our tests (Table 1); however, luliconazole is cytotoxic at low concentrations, prohibiting its systemic use as an antifungal [14].

Alexidine, another topical drug, also showed anti-*Sporothrix* activity. Initially developed as an anticancer drug, it is now used as an antibacterial agent in oral rinse and contact lens solutions [17]. The antifungal activity of alexidine was previously shown against *Candida* spp., *Aspergillus fumigatus*, and *Cryptococcus neoformans* [18]. Here, we demonstrated that alexidine exhibits in vitro activity against dimorphic fungi.

Rubitecan is an antitumoral drug that inhibits DNA topoisomerase I [19]. This camptothecin derivative inhibits in vitro HIV replication in different cell types [20]. The antifungal activity of rubitecan was not previously reported, and our results showed that only *S. brasiliensis* and *S. schenckii* were susceptible to this compound in concentrations lower than 1 µM (Table 1).

Similar to rubitecan, it was possible to observe that some library compounds that inhibited *S. brasiliensis* growth were not active against *S. schenckii* or *S. globosa* (Table 1). According to our previous experience, *S. brasiliensis* is usually more susceptible during in vitro evaluation than other *Sporothrix* species [21]. Therefore, *S. brasiliensis* was selected for our initial screening with the 400 compounds. In general, the three species showed a distinct activity profile. It was interesting to note, for example, that the new azoles isavuconazole and ravuconazole were more active against *S. globosa*, with lower MIC values than for *S. brasiliensis* and *S. schenckii* (Table 1).

Regarding new compounds (named with “MMV” initials), none of the eight molecules identified as active against *S. brasiliensis* was able to inhibit all three *Sporothrix* species (Table 1). MMV identified MMV1634360 and MMV1634491 as antifungal compounds [10], and our results confirm their ability to inhibit fungal growth. Some of these compounds were described as exhibiting activity against other fungi. MMV1634491 showed in vitro antifungal activity against mycetoma agents and improved the survival rate of *Galleria mellonella* infected with *Madurella mycetomatis*, as well as the compound MMV019724 (previously described as an antiviral molecule [10]) [22]. MMV019724 inhibited the fungal growth of *Cryptococcus* spp. and *Candida auris* at 5 µM; however, it showed cytotoxicity at this concentration against RAW 264.7 macrophages [23].

We selected olorofim for further experiments due to its great in vitro activity against the three *Sporothrix* species and as a new antifungal already involved in phase III of human tests [24]. Olorofim (formerly F901318, F2G Ltd., Princeton, NJ, USA) is being evaluated as an oral treatment for life-threatening systemic fungal infections, including invasive aspergillosis, invasive scedosporiosis, invasive lomentosporiosis, coccidioidomycosis, infections due to the *Scopulariopsis* species, and invasive fusariosis. In 2022, olorofim commercialization was licensed in Asia and Europe [15,25].

The promising in vitro activity of olorofim was already reported for several species of filamentous fungi, with MIC values ranging from 0.03 to 1 µg/mL (0.06 to 2 µM) for *Aspergillus* spp., *Scedosporium* spp., and *Fusarium* spp., for example [15]. Olorofim also exhibited potent activity against the dimorphic fungi *Blastomyces dermatitidis*, *Coccidioides immitis*, and *Histoplasma capsulatum*. However, it has no action against the medically relevant yeasts *Candida* spp. and *Cryptococcus* spp. [26].

According to the literature, olorofim displayed a fungistatic action profile [27]; however, in our work, we showed a fungicidal effect at 10 µM against the three *Sporothrix* pathogenic species (Figure 2B). Olorofim inhibited 50% or more of the metabolic activity of the *Sporothrix* mature biofilms at concentrations lower than 4 µM (Figure 2C). It is noteworthy that olorofim could not inhibit the mature biofilms of *Aspergillus fumigatus* and *Lomentospora prolificans* [28]. It was also verified that *S. globosa* was more sensitive to olorofim, since its yeasts and biofilms were more susceptible to lower concentrations than against *S. brasiliensis* and *S. schenckii*.

In addition to exhibiting great antifungal activity and selectivity against *Sporothrix* pathogenic species, olorofim induced profound alterations on the cell surface and cell cycle arrest in *S. brasiliensis* yeasts (Figure 3). Olorofim inhibits the biosynthesis of pyrimidine due to the reversible inhibition of the enzyme dihydroorotate dehydrogenase (DHODH) located in the mitochondria, causing a reduction in uridine-5′-monophosphate (UMP) and uridine-5′-triphosphate (UTP). UTP plays a central role in several metabolic pathways in eukaryotic cells; therefore, its reduction induces many disturbances inside the cell, such as: (i) a decrease in DNA replication because UTP is necessary to form DNA pyrimidine derivatives; (ii) the impairment of protein synthesis due to decreased RNA levels; and (iii) alterations on fungal cell walls due to the UTP requirement for the generation of UDP–sugars, which are substrates for glucans and chitin biosynthesis [15]. UTP can also be converted into cytidine triphosphate (CTP), which acts in phospholipid synthesis [29]. Thus, olorofim can also disturb the plasma membrane.

We observed a dose-dependent DNA accumulation in *S. brasiliensis* yeasts with exposure to olorofim, indicating cell cycle arrest probably due to the inhibition of mitosis, as reported with *A. fumigatus* [30]. Olorofim also induced cell wall remodeling with increased chitin and mannans, with the latter exhibiting the most remarkable changes, whereas β-glucan was decreased, as observed with *A. fumigatus* [30] (Figure 3). The *Sporothrix* cell wall is externally coated by a peptido–rhamnomannans layer [31], which is visualized in Figure 3(Hiv) as the electron-dense layer forming the cell wall. Untreated yeasts showed a lower peptido-rhamnomannans layer as visualized by transmission electron microscopy (Figure 3(Hi)), confirming the higher fluorescent stain with concanavalin A. The increase in the cell wall thickness observed due to olorofim treatment confirmed the fluorimetry results with concanavalin A and calcofluor white.

**Figure 5:**
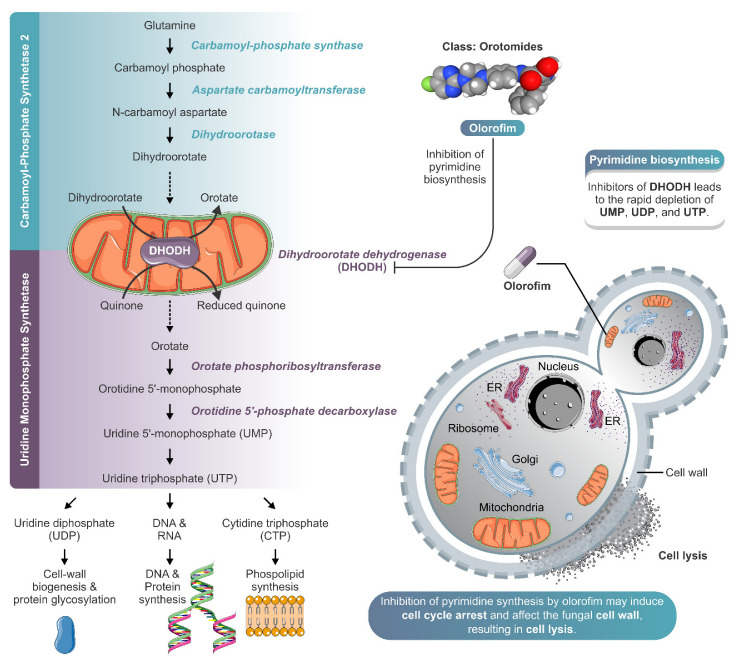
Schematic model of olorofim action against *Sporothrix* yeasts. Olorofim belongs to a new class of antifungal agents called the orotomides, which inhibits fungal pyrimidine biosynthesis. Olorofim inhibits dihydroorotate dehydrogenase, an enzyme of de novo pyrimidine biosynthesis, leading to the rapid depletion of uridine 5′-monophosphate (UMP), uridine diphosphate (UDP), and uridine triphosphate (UTP). The reduction in UTP levels can cause a decrease in cell wall biogenesis, protein glycosylation, and synthesis of DNA, protein, and phospholipid. In *Sporothrix* yeast cells, the inhibition of pyrimidine synthesis by olorofim may induce cell cycle arrest and affect the fungal cell wall, resulting in cell lysis. The illustration was partially based on Servier Medical Art elements and licensed under a Creative Commons Attribution 3.0 Unported License. ER: endoplasmic reticulum.

The cell wall also presented a granular appearance after olorofim exposure, which was corroborated by the decrease in cellular conductance. Cell wall remodeling modified the physicochemical properties of yeasts, reducing their electronegativity and conductance (properties related to cell surface aspects). These changes can compromise the ability of yeasts to adhere to a substrate. Based on our results, we hypothesize that olorofim inhibits dihydroorotate dehydrogenase in *Sporothrix* cells by blocking pyrimidine biosynthesis (Figure 5). We verified that these profound morphophysiological alterations in *S. brasiliensis* yeasts impaired their ability to adhere to keratinocytes (Figure 4). Further in vivo experiments should be performed to confirm the potential of olorofim in sporotrichosis treatment.

## 5. Conclusions

In summary, after screening the Pandemic Response Box library, we identified olorofim with strong in vitro anti-*Sporothrix* activity. Olorofim was effective against the main pathogenic *Sporothrix* species (*S. brasiliensis*, *S. globosa*, and *S*. *schenckii*), inducing cell cycle arrest and cell wall remodeling and exhibiting antibiofilm effects. Taken together, our results indicate that olorofim is a promising new antifungal against sporotrichosis agents.

## Figures and Tables

**Table 1:** Inhibitory effects of twenty-four compounds on the pathogenic *Sporothrix* species.

Compounds	Minimum Inhibitory Concentration (µm) ^a^		
	*S. brasiliensis*	*S. globosa*	*S. schenckii*
**Reference antifungal**			
Itraconazole	0.125	0.03	0.25
**Antifungals**			
**Commercial drugs**			
Abafungin	0.5	0.5	1
Amorolfine	0.125	0.5	1
Butenafine	0.25	1	0.25
Ciclopirox	0.5	0.03	1
Deferasirox	0.5	0.5	>1
Eberconazole	0.125	0.03	0.5
Isavuconazonium	0.5	0.03	0.5
Ketoconazole	0.25	0.125	0.125
Luliconazole	0.004	0.004	0.004
Miconazole	0.125	0.03	0.125
Ravuconazole	1	0.03	0.125
Terbinafine	0.5	0.5	0.125
**New drugs ^b^**			
Olorofim	0.06	0.03	0.06
**New molecules**			
MMV1634360	1	>1	0.25
MMV1634491	1	0.03	>1
**Antibacterials**			
**Commercial drugs**			
Alexidine	1	0.5	0.25
**New molecules**			
MMV1579784	0.5	>1	>1
MMV1579786	0.25	0.03	>1
MMV1579788	0.5	>1	>1
MMV1782140	0.25	>1	1
**Antivirals**			
**New drugs ^b^**			
Rubitecan	0.25	>1	1
**New molecules**			
MMV019724	0.25	>1	1
MMV642550	0.25	>1	>1

^a^ Minimum inhibitory concentration was defined as the concentration that inhibited at least 50% of fungal growth. ^b^ Drugs in clinical trials.

**Table 2:** Cytotoxicities of olorofim and itraconazole.

Compounds	*Sporothrix* spp.	HaCaT	RAW 264.7	Selectivity Index
	MIC_median_ (µm)	CC_50_ (µm)	CC_50_ (µm)	
**Olorofim**	0.06	>100	>100	>1666.7
**Itraconazole**	0.125	>100	>100	>800

## Data Availability

The data in this study are available in the presented manuscript.

## Supplementary Materials

The following supporting information can be downloaded at: https://www.mdpi.com/article/10.3390/jof8101004/s1, Table S1: Inhibition of *Sporothrix brasiliensis* ATCC MYA 4823 in yeast phase after exposure to 1 μM of the Pandemic Response Box compounds.
