# Supplementary material for: Screening of Pandemic Response Box Library Reveals the High Activity of Olorofim against Pathogenic Sporothrix Species
Source: J Fungi (Basel). 2022 Sep 25;8(10):1004. doi: 10.3390/jof8101004 (PMC9605001; doi:10.3390/jof8101004)
Supplement: Supplementary file 1 [file jof-08-01004-s001.zip › jof-1906899-supplementary.pdf]

Table S1. Inhibition of *Sporothrix brasiliensis* ATCC MYA 4823 in yeast phase after exposure to 1 μM of the Pandemic Response Box compounds.

Pandemic Response Box Compounds	%Inhibition*	Pandemic Response Box Compounds	%Inhibition*
MMV1634492	98	MMV689401	27
MMV1782108	14	MMV1782351	7
MMV1634494	100	MMV1782387	6
MMV1634386	24	MMV1782389	11
MMV637528	100	MMV1782388	3
MMV1782109	27	MMV1634557	13
MMV1645051	18	MMV030787	68
MMV1634491	93	MMV000028	45
MMV002731	96	MMV002722	100
MMV000059	21	MMV1593535	12
MMV637104	100	MMV1593533	4
MMV637533	100	MMV422940	3
MMV1634362	100	MMV1582497	11
MMV1634360	79	MMV1581553	16
MMV002565	16	MMV1581548	10
MMV396785	100	MMV1580850	54
MMV003143	9	MMV002740	12
MMV002337	10	MMV1579783	30
MMV1634363	11	MMV1578925	57
MMV1634359	100	MMV1578889	19
MMV1782110	30	MMV1578572	21
MMV1634493	100	MMV1578568	20
MMV344625	15	MMV1580173	16
MMV1634358	81	MMV1634403	10
MMV1578560	6	MMV1634390	11
MMV640014	79	MMV1633967	14
MMV004508	43	MMV1593534	16
MMV1782212	10	MMV1582498	12
MMV1782227	11	MMV1582494	27
MMV1782226	8	MMV1582493	61
MMV1782221	11	MMV000043	29
MMV1782224	100	MMV003137	46
MMV1782223	16	MMV1581552	57
MMV1782218	21	MMV1581551	13
MMV1782217	24	MMV1580853	10
MMV1782216	30	MMV1580841	8
MMV1009286	21	MMV1579878	14
MMV1782225	64	MMV1579845	21
MMV1782354	100	MMV1579787	27

*Mean of three independet experimets performed in duplicate.

Continuation of Table S1.

Pandemic Response Box Compounds	%Inhibition*	Pandemic Response Box Compounds	%Inhibition*
MMV1579784	80	MMV002459	16
MMV1483032	13	MMV637945	4
MMV508427	4	MMV1580854	1
MMV1578565	6	MMV1580851	1
MMV1578561	21	MMV1579849	1
MMV1578557	12	MMV1579844	48
MMV000725	13	MMV141011	41
MMV1634384	13	MMV002516	6
MMV1634383	24	MMV688755	21
MMV1613560	23	MMV020752	15
MMV1230557	7	MMV1229204	5
MMV1593540	0	MMV1578575	13
MMV1593539	3	MMV1578556	1
MMV1582495	5	MMV1663457	9
MMV1582487	7	MMV1341773	3
MMV021759	5	MMV1633970	7
MMV1581555	8	MMV1633965	5
MMV1581547	12	MMV093250	13
MMV687800	11	MMV1593541	2
MMV1580848	13	MMV1593537	13
MMV1580842	27	MMV1582492	11
MMV1580840	12	MMV1582491	8
MMV046261	11	MMV1582382	10
MMV1579846	12	MMV002354	5
MMV1578842	0	MMV1581557	10
MMV1579776	9	MMV1581554	46
MMV1579775	8	MMV1581550	14
MMV1579354	10	MMV1581546	16
MMV1578899	12	MMV1580844	29
MMV1578890	12	MMV1579788	87
MMV1578886	10	MMV1579785	12
MMV1578573	22	MMV811071	18
MMV1578571	26	MMV1578898	7
MMV687273	24	MMV1549626	29
MMV1634387	28	MMV1578559	31
MMV1633966	29	MMV1578579	31
MMV1505642	20	MMV1578578	30
MMV1593542	21	MMV1578574	23
MMV1593538	20	MMV1578569	20

*Mean of three independet experimets performed in duplicate.

Continuation of Table S1.

Pandemic Response Box Compounds	%Inhibition*	Pandemic Response Box Compounds	%Inhibition*
MMV1578563	21	MMV1579847	10
MMV1578558	11	MMV1579782	1
MMV1782140	83	MMV1579777	3
MMV1634391	18	MMV292173	5
MMV1633674	22	MMV102270	5
MMV002169	7	MMV000008	11
MMV002612	13	MMV002260	7
MMV1613562	18	MMV102833	18
MMV689758	34	MMV565773	52
MMV1593532	20	MMV1578577	9
MMV002224	14	MMV233495	10
MMV637659	5	MMV002676	3
MMV1580846	12	MMV1633675	3
MMV1580845	14	MMV303733	3
MMV1579850	10	MMV003249	0
MMV374187	8	MMV001438	2
MMV1579781	11	MMV1582496	1
MMV1579780	5	MMV1582490	10
MMV688756	14	MMV1582489	1
MMV1578897	22	MMV1582488	11
MMV001014	25	MMV002665	10
MMV1578885	19	MMV1581559	11
MMV687696	9	MMV1581545	0
MMV011565	8	MMV1580855	2
MMV452821	10	MMV1580852	4
MMV1578576	8	MMV1580849	10
MMV1578562	12	MMV1580839	4
MMV1578555	10	MMV1579779	3
MMV1578554	9	MMV1579778	2
MMV1634402	8	MMV099714	3
MMV1634399	42	MMV1578891	0
MMV000051	13	MMV124656	12
MMV002287	30	MMV975972	17
MMV831201	21	MMV1578884	13
MMV687801	11	MMV108465	9
MMV1593531	17	MMV1480967	8
MMV1581558	13	MMV1578570	11
MMV1581556	14	MMV1578564	17
MMV1581549	10	MMV1782215	8

*Mean of three independet experimets performed in duplicate.

Continuation of Table S1.

Pandemic Response Box Compounds	%Inhibition*	Pandemic Response Box Compounds	%Inhibition*
MMV1782402	10	MMV639951	46
MMV1782411	15	MMV1580491	10
MMV214956	25	MMV1580492	11
MMV1634395	11	MMV690621	8
MMV1634071	14	MMV1782112	0
MMV1633962	19	MMV1782107	18
MMV1633678	23	MMV1782106	26
MMV690480	19	MMV1782105	28
MMV1593517	29	MMV1782104	38
MMV1593515	21	MMV1782101	24
MMV1581032	24	MMV1633969	3
MMV1580799	4	MMV596723	0
MMV001726	14	MMV1581378	0
MMV690653	16	MMV1581031	0
MMV1580484	10	MMV1580797	1
MMV1580478	14	MMV001761	1
MMV1782102	11	MMV002015	7
MMV394033	20	MMV056052	16
MMV1634404	20	MMV1580504	26
MMV1634388	20	MMV001961	30
MMV180402	27	MMV003738	34
MMV642550	83	MMV690540	38
MMV1580798	13	MMV614278	25
MMV1580800	16	MMV1580489	15
MMV1580503	17	MMV1580499	14
MMV690547	15	MMV1782115	14
MMV1165877	12	MMV1782114	19
MMV1580497	23	MMV018362	17
MMV1580495	21	MMV1634556	24
MMV1645152	23	MMV1634401	31
MMV1633677	29	MMV1634394	43
MMV1593520	26	MMV1633964	45
MMV1581029	31	MMV1593516	43
MMV1580794	23	MMV1593513	43
MMV001793	22	MMV1593511	43
MMV251679	17	MMV637306	42
MMV003291	20	MMV1581377	43
MMV009948	27	MMV002780	40
MMV1174026	18	MMV1580801	45

*Mean of three independet experimets performed in duplicate.

Continuation of Table S1.

Pandemic Response Box Compounds	%Inhibition*	Pandemic Response Box Compounds	%Inhibition*
MMV637879	46	MMV1634389	34
MMV617332	15	MMV1581035	3
MMV1580480	8	MMV1593544	3
MMV1580494	12	MMV1593521	6
MMV275100	13	MMV1593514	5
MMV1782103	20	MMV1581036	6
MMV1782098	17	MMV1581030	15
MMV1634397	22	MMV247764	14
MMV1633963	27	MMV019724	90
MMV1593519	17	MMV638198	23
MMV002505	17	MMV637413	8
MMV1581034	11	MMV658803	6
MMV1581033	12	MMV1580500	7
MMV1557856	14	MMV1580498	2
MMV1580796	88	MMV1580488	30
MMV1580505	20	MMV1580486	10
MMV687798	23	MMV1782222	12
MMV003297	20	MMV690706	24
MMV1580487	26	MMV1782220	16
MMV1580485	26	MMV1782213	20
MMV098836	43	MMV1782214	14
MMV218827	12	MMV1782211	36
MMV1782111	18	MMV1782210	18
MMV1782097	20	MMV1782208	20
MMV1634398	25	MMV1006203	17
MMV1634396	17	MMV1782353	14
MMV1634385	23	MMV1782349	19
MMV1633968	25	MMV1782355	17
MMV688991	19	MMV1782352	9
MMV1580502	44	MMV1782350	19
MMV637855	30	MMV1782386	29
MMV690467	32	MMV1782412	31
MMV1580493	47	MMV1613563	31
MMV1580482	35	MMV1580843	38
MMV1580501	28	MMV690555	21
MMV1580496	35	MMV1613559	13
MMV1580490	30	MMV1634361	17
MMV1782113	31	MMV002350	18
MMV1634393	30	MMV1580483	20

*Mean of three independet experimets performed in duplicate.
